# Investigation of an EPID‐based dose verification system for patient‐specific quality assurance in a harmony device

**DOI:** 10.1002/acm2.70201

**Published:** 2025-08-21

**Authors:** Dohyeon Yoo, Hyunggun Lee, Sangmin Lee, Soorim Han, Taeho Kim, Changhwan Kim, Min Cheol Han, Jin Sung Kim

**Affiliations:** ^1^ Department of Radiation Oncology, Yonsei Cancer Center, Yonsei University, College of Medicine & Heavy Ion Therapy Research Institute Yonsei University College of Medicine Seoul South Korea; ^2^ Korea Institute of Radiological and Medical Sciences Seoul South Korea

**Keywords:** harmony pro, IMRT, patient‐specific quality assurance, RadCalc, VMAT

## Abstract

**Background:**

Precise patient‐specific quality assurance (PSQA) is critically important in advanced treatments.

**Purpose:**

This study aimed to evaluate the performance of the RadCalc software's electronic portal imaging device (EPID)‐based patient specific QA function, focusing on its ability to accurately reconstruct doses from EPID images acquired on the Elekta Harmony Pro linear accelerators (LINACs).

**Methods:**

Beam modeling was conducted for 6‐MV and 6‐MV flattening filter–free (FFF) photon beams on the recently released Harmony Pro LINAC system in RadCalc. Volumetric modulated arc therapy (VMAT) plans for 6‐MV (19 patients) and 6‐MV FFF (10 patients) were generated using the ArcCHECK phantom to replicate clinical treatments. Dose calculations from RadCalc, using portal images, were compared to those generated by the treatment planning system (TPS) through gamma analysis at 1%/1, 2%/2, and 3%/3 mm, with a 90% minimum passing rate.

**Results:**

For 6‐MV and 6‐MV FFF beams, gamma analysis comparing EPID data with RadCalc showed a 98.83% passing rate at 2%/2 mm. For VMAT plans, 6‐MV beams achieved the highest passing rate (99.88%) for the breast case at 3%/3 mm and the lowest (82.33%) for the lymphatic node case. Similarly, 6‐MV FFF beams yielded the highest passing rate (99.87%) for the abdominal case at 3%/3 mm and the lowest (94.07%) for the bone case.

**Conclusions:**

RadCalc showed high accuracy for dose evaluation on the Elekta Harmony Pro LINAC. Beam modeling achieved a gamma passing rate of at least 98% (2%/2 mm), demonstrating reliable performance even under minor setup variations.

## 1 INTRODUCTION

Modern radiation therapy techniques, such as intensity‐modulated radiation therapy (IMRT) and volumetric modulated arc therapy (VMAT), utilize mutileaf collimators (MLCs) to shape radiation beams with high precision. This enables the delivery of conformal doses to the target area while minimizing exposure to surrounding healthy tissues.^1^, ^2^, ^3^, ^4^, ^5^, ^6^, ^7^ As these treatment methods become increasingly complex, patient‐specific quality assurance (PSQA) is critical to ensure accurate dose delivery.^8^ PSQA methods are generally categorized into measurement‐ and calculation‐based approaches.^9^, ^10^, ^11^, ^12^

Measurement‐based PSQA directly measures the radiation dose delivered to a phantom that mimics a patient's anatomy. This approach typically employs a water‐equivalent phantom, with dose measurements obtained using ionization chambers, radiosensitive films, or detector arrays. Although measurement‐based PSQA reliably measures the dose delivered to a phantom, it has inherent limitations. One main limitation is that the treatment plan is delivered to a homogeneous phantom, which fails to replicate tissue heterogeneity in actual patients.^13^, ^14^ Conversely, calculation‐based PSQA employs independent dose verification algorithms to evaluate dose distribution, irrespective of whether a phantom setup is used.^15^, ^16^, ^17^, ^18^, ^19^ This method calculates the delivered dose distribution directly on the patient's computed tomography (CT) image using dose calculation algorithms such as the collapsed cone and Monte Carlo algorithms and by incorporating machine data—such as jaw and MLC positions, and monitor units (MUs) for each control point. These data are either extracted from device logs or reconstructed from the exit dose measured using an electronic portal imaging device (EPID). By integrating such data, calculation‐based PSQA can account for tissue heterogeneity. The accuracy of calculation‐based PSQA largely relies on the precision of beam modeling and the reliability of the dose calculation algorithm within the intermediate dose calculation (IDC) system, especially if log data or EPID measurements are considered clinically reliable.

Recent advancements in IDC algorithms have significantly improved the accuracy of calculation‐based PSQA methods by independently verifying the dose calculated by a treatment planning system (TPS).^20^, ^21^, ^22^ This improvement, combined with integrating patient log data and EPID information, has facilitated the development of more efficient and widely adopted PSQA workflows in clinical practice. Consequently, various software solutions have been introduced to streamline PSQA processes, including EpiGray (DOSIsoft, France), Mobius3D (Varian Medical Systems, USA), PerFRACTION (Sun Nuclear Corporation, USA), and RadCalc (LAP, Germany), each offering unique functionalities, such as EPID‐based dose verification and log file analysis.^23^, ^24^, ^25^, ^26^, ^27^, ^28^, ^29^, ^30^, ^31^, ^32^

When the linear accelerator (LINAC) and PSQA software are from the same manufacturer (e.g., TrueBeam and Mobius3D), the setup process is typically more seamless due to the manufacturer's extensive experience integrating its products. However, additional configuration steps and considerations may be necessary, when integrating heterogeneous systems from different manufacturers, from setup to clinical implementation.^33^, ^34^ While existing PSQA methods—especially those involving same‐vendor integration—offer streamlined workflows, they may not be directly applicable when employing software and hardware from different vendors. This mismatch can pose challenges in modeling accuracy, data communication, and dose reconstruction fidelity. Therefore, validating the clinical applicability of RadCalc for use with the Elekta Harmony Pro LINAC, which represents a heterogeneous integration scenario, become essential.

Our institution recently completed the installation of the latest LINAC model by Elekta, Harmony Pro, integrated with RadCalc. In this study, we present our experience with the clinical setup, implementation process, and key considerations for integrating the system into clinical practice. Despite some potential setup errors, the findings suggest that RadCalc is a reliable tool for PSQA. The results indicate that the system is clinically applicable and can effectively verify complex dose distributions, enhancing the overall safety and effectiveness of radiation treatments in clinical practice. However, future studies should be conducted to refine the beam modeling to improve accuracy and enhance the overall performance of the system.

Our study aimed to validate the accuracy of the RadCalc, an EPID‐based PSQA system for Elekta Harmony Pro linear accelerators (LINACs).

## 2 MATERIALS AND METHODS

### 2.1 Harmony pro

**FIGURE 1:**
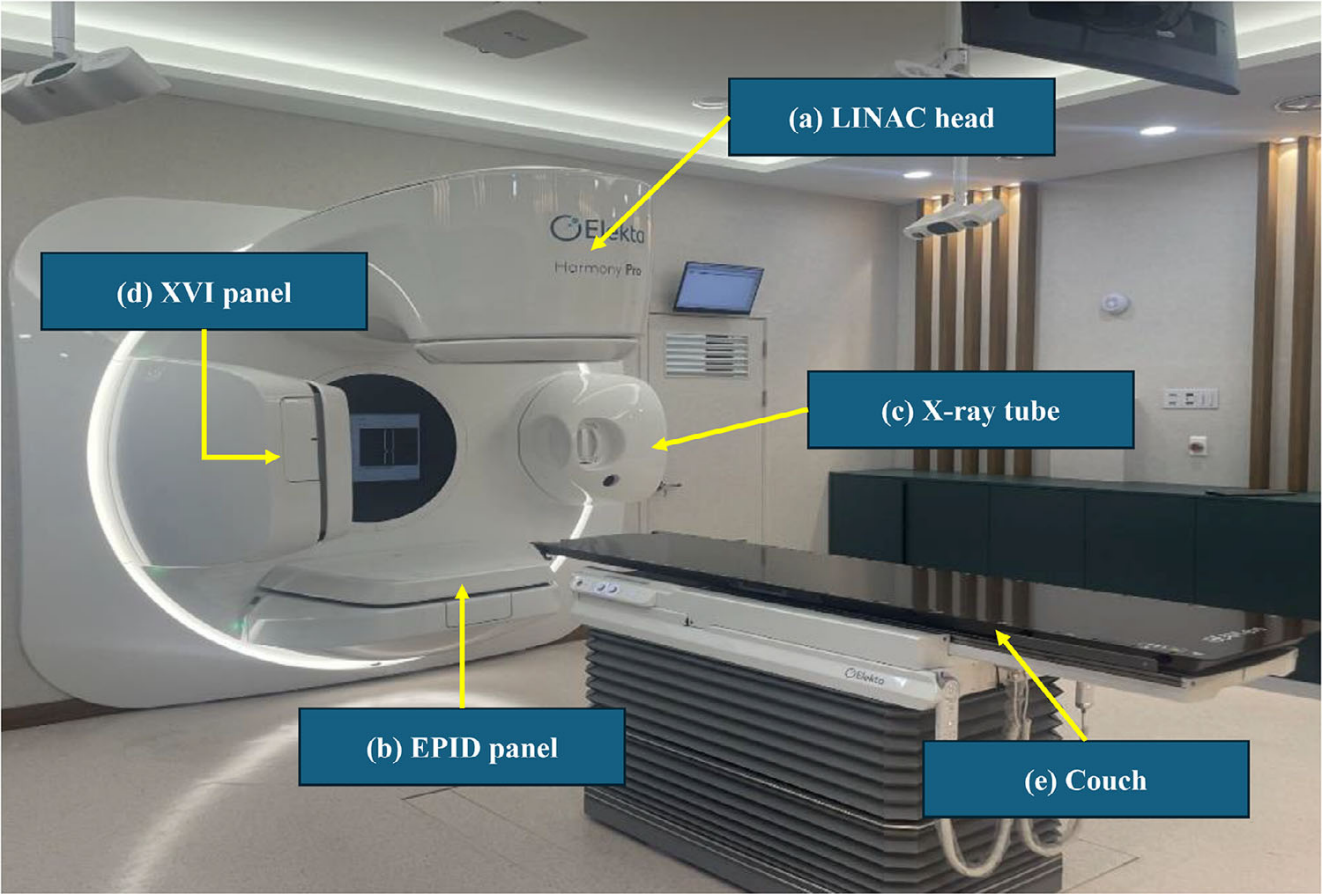
Overview of the Harmony Pro (Elekta, Stockholm, Sweden) at the Yonsei Cancer Center: (a) Linear accelerator (LINAC) head, (b) electronic portal imaging device (EPID), (c) X‐ray tube, (d) X‐ray volumetric imaging (XVI), and (e) couch.

The Harmony Pro is the latest LINAC manufactured by Elekta, officially released on September 9, 2020. This system can deliver 6‐MV and 6‐MV flattening filter‐free (FFF) photon beams. Figure 1 shows the Harmony Pro system installed at our institute. It features a maximum field size of 40 × 40 cm^2^, defined by its advanced MLCs. The MLC system comprises 160 interdigitating tungsten leaves, each with a nominal leaf width projection of 5 mm at the isocenter, allowing for high‐resolution beam shaping. The leaves can travel up to 15 cm across the central axis and achieve speeds of up to 6.5 cm/s when used with a dynamic leaf guide, thereby enabling efficient modulation for complex treatment plans. The Harmony Pro features Elekta Rubicon optical positioning system, ensuring submillimeter leaf positioning accuracy and transmission levels below 0.375%. Additionally, the system provides an isocenter clearance of 45 cm, enhancing patient positioning flexibility and optimizing treatment delivery. Furthermore, the Harmony Pro supports advanced image‐guided radiation therapy using 2D, 3D, and 4D imaging, enabling precise target localization during treatment. Moreover, the EPID panel was combined with the Elekta iViewGT software, which enables automated image acquisition, analysis, and comparison with the reference images acquired from treatment planning. Earlier Elekta LINAC models, such as Infinity and VersaHD, were equipped with iViewGT Version 3.4. Conversely, the Harmony Pro features the latest version iViewGT 3.5, which offers improved image processing algorithms, enhanced system stability, and faster data transfer.^35^

### 2.2 RadCalc software

#### 2.2.1 Introducing RadCalc

RadCalc (LAP, Germany, Version 7.3) is a comprehensive software for advanced dose verification in radiation therapy. It supports collapsed cone convolution (CCC) and Monte Carlo (MC) algorithms, enabling high‐precision dose calculations. By utilizing EPID images obtained from a LINAC, RadCalc performs a three‐step indirect back projection by deconvolving the EPID images to account for patient and panel scatter, reconstructing the machine parameters by back projecting the resulting fluences and reconstructing the dose distribution on the corresponding CT images (slice thickness of 3 mm) with Collapsed Cone.^36^

The RadCalc operates in three distinct modes: (1) pretreatment verification, (2) in vivo, and (3) fractional machine quality assurance (QA). In the pretreatment mode, EPID images are captured before treatment, with no physical phantom placed between the radiation source and the detector. This mode is primarily used to verify treatment plans and assess the accuracy of dose delivery in the pre‐treatment phase. The in vivo mode leverages EPID images captured during each actual patient treatment session to verify the dose delivered for that particular fraction. The fractional machine QA mode uses delivery data from patient treatment sessions to assess machine performance.

We used RadCalc (Version 7.3) with the CCC algorithm, focusing on the pretreatment mode in this study; all comparisons were conducted using EPID images. We configured and tested the system exclusively under this setup with the Harmony Pro. This approach enabled us to assess the accuracy and feasibility of using an EPID for pretreatment QA. The successful implementation of RadCalc requires a systematic setup that involves three main steps—beam modeling for the CCC algorithm, independent EPID modeling, and validation procedures using various PSQA cases. These two modeling processes were conducted in collaboration with an LAP specialist. The details of each process are discussed in the following sections.

#### 2.2.2 Beam modeling

Before using the RadCalc software, a comprehensive beam‐modeling process was conducted to ensure accurate PSQA. The EPID beam modeling focused on 6‐MV and 6‐MV FFF photon beams. This process involved inputting measured beam data into the RadCalc software to create a reliable beam model. The software incorporated the percentage depth dose (PDD), tissue phantom ratio (TPR), beam profiles, and output factors. Notably, since the RadCalc software allows users the flexibility to define reference conditions such as source‐to‐surface distance (SSD) or source‐to‐axis distance (SAD) parameters, this study utilized measurement data extracted directly from the radiation TPS (RayStation, RaySearch Laboratories, Sweden, Version 11B) in our institute to ensure consistency and accuracy.

#### 2.2.3 EPID modeling

**FIGURE 2:**
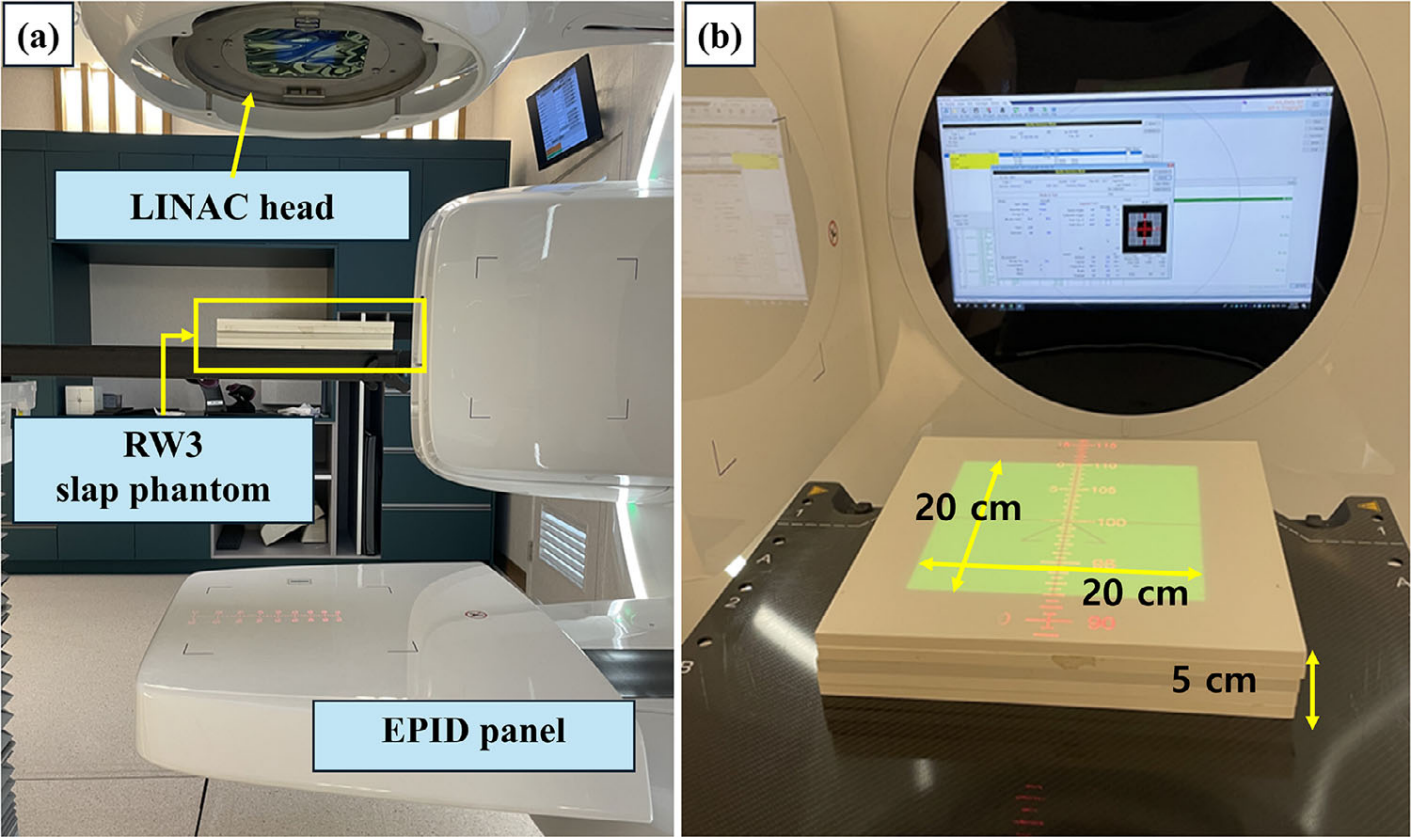
(a) Experimental setup for electronic portal imaging device (EPID) modeling on the Harmony Pro with an RW3 slab phantom positioned on the couch. (b) Detailed experimental setup for EPID modeling with a 20 × 20 cm2 field size and an RW3 slab phantom at a depth of 5 cm (The surface‐to‐source distance (SSD) is 100 cm as shown; all EPID modeling and dose reconstructions were performed using a source‐to‐axis distance [SAD] of 100 cm).

Following beam modeling, EPID modeling was conducted. Beam measurement data for the EPID modeling were obtained from EPID images captured by irradiating beams of various field sizes (3 × 3, 5 × 5, 10 × 10, 15 × 15, and 20 × 20 cm^2^) onto an RW3 slab phantom (PTW, Germany) of varying thicknesses (0, 5, 10, 15, 20, and 25 cm) at an SAD of 100 cm. Figure 2 shows the setup for EPID modeling with 6‐MV and 6‐MV FFF photon beams using a 20 × 20 cm^2^ field. Although the source‐to‐surface distance (SSD) is 100 cm as shown, all EPID modeling and dose reconstruction were performed using a source‐to‐axis distance (SAD) of 100 cm. Using these EPID image data, the deconvolution kernel for PSQA calculations was generated using the RadCalc software. To verify the implementation of the EPID modeling results, the EPID data obtained from the 20 × 20 cm^2^ field were used in RadCalc to calculate the dose on the RW3 phantom.^37^ Although the modeling was not performed with a CIRS phantom, the measurements using RW3 slab phantoms of varying thicknesses (0–25 cm) followed the commissioning procedure recommended by the LAP specialist engineer, allowing reliable modeling. The reconstructed dose distribution was compared with the reference dose calculated by TPS (RayStation, CCC algorithm) under identical conditions using gamma analysis, which was performed using 1%/1 mm and 2%/2 mm criteria and served as a validation step for confirming the accuracy of the EPID deconvolution kernel prior to its application in clinical VMAT plans.

#### 2.2.4 PSQA validation using VMAT plans

**FIGURE 3:**
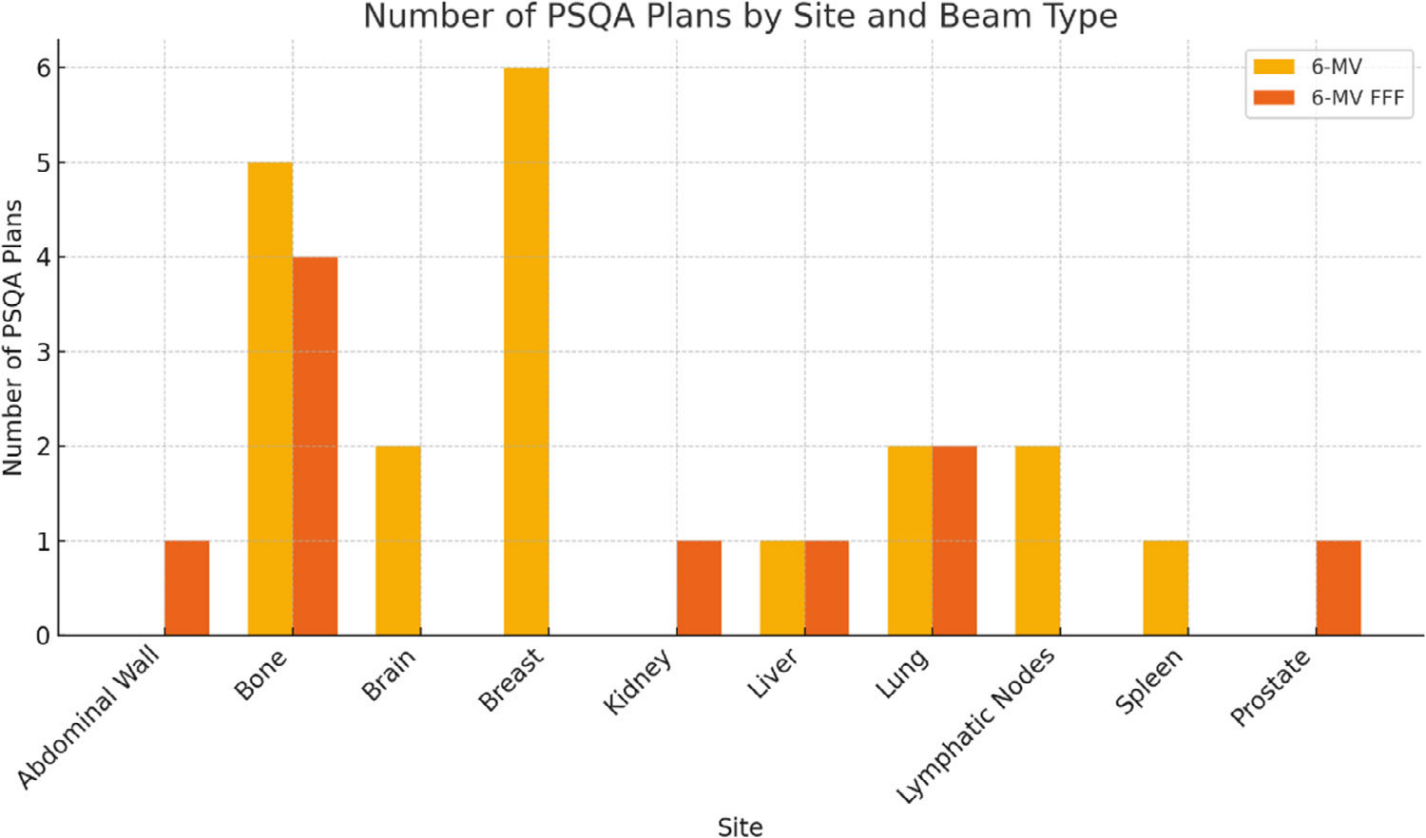
Detailed treatment plan information for selected VMAT plans for 6‐MV and 6‐MV FFF photon beams.

PSQA cases were designed to replicate actual treatment plans to assess the accuracy of the CCC algorithm and its modeling. These cases consisted of VMAT plans designed for 6‐MV and 6‐MV FFF beams, encompassing a range of treatment sites. A total of 29 cases were analyzed (Figure 3). Each treatment plan was transferred to the ArcCHECK phantom (Sun Nuclear, Melbourne, FL, USA) and subsequently imported into the RadCalc software for IDC and pretreatment calculations. The ArcCHECK measurements were analyzed using SNC Patient software (Sun Nuclear Corporation, version 8.5.1).

The accuracy of the beam modeling and CCC algorithm was initially assessed by conducting dose calculations in RadCalc using only the IDC system without incorporating EPID data. These calculated dose distributions were then compared to those generated by the TPS using gamma analysis.

**FIGURE 4:**
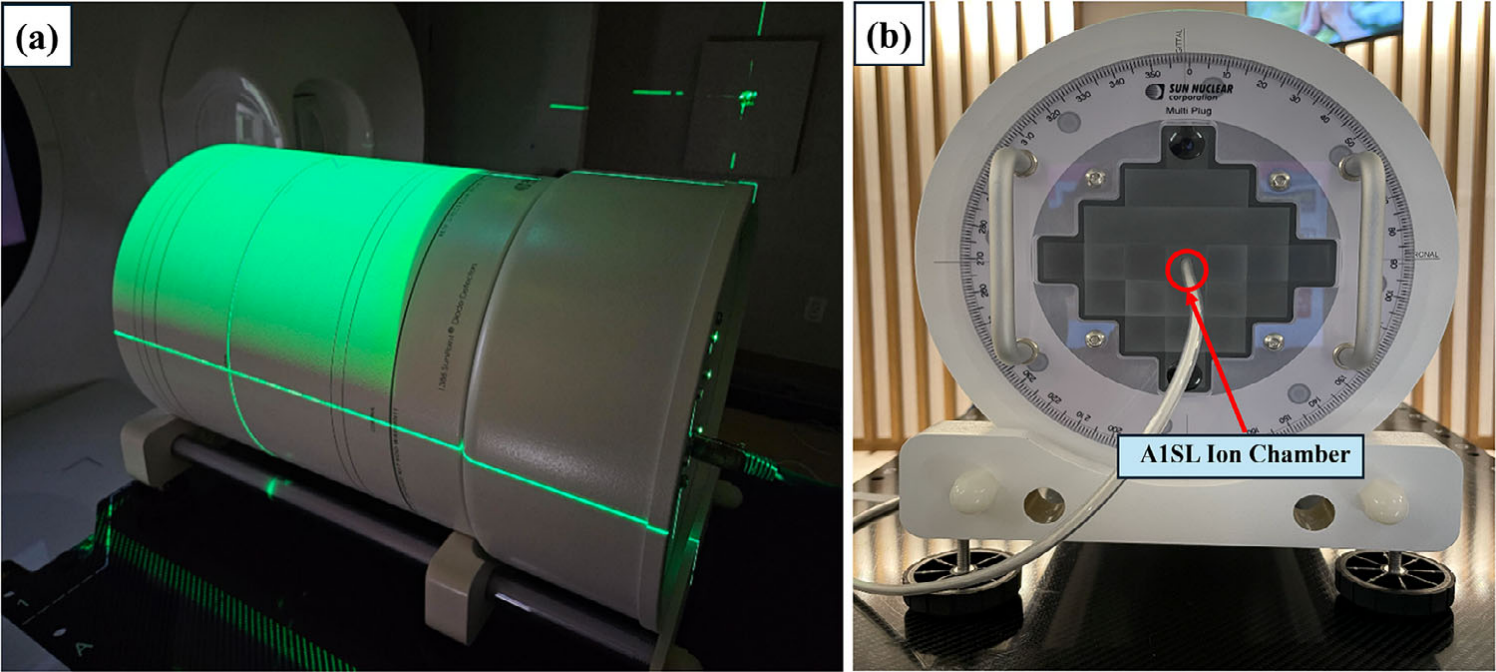
(a) ArcCHECK phantom laser‐aligned for measurements. (b) A1SL ion chamber positioned at the ArcCHECK center to measure the dose at the isocenter.

Subsequently, pretreatment calculations were conducted in RadCalc using EPID images obtained during actual beam delivery. To validate these calculations quantitatively, measurement‐based PSQA was conducted using the ArcCHECK phantom and an A1SL ion chamber (Standard Imaging, Middleton, WI, USA) positioned at the isocenter (Figure 4). The measured results were compared to RadCalc calculations using point‐dose comparisons and gamma passing rates. In this study, all the plans used for validation demonstrated gamma passing rates greater than 95% (minimum 98%) upon comparing the ArcCHECK measurements with TPS calculations. For all analyses, gamma index criteria were set at 2%/2 mm and 3%/3 mm, with a passing threshold of >90%. The point‐dose error threshold was set to be <5%.^38^ To maintain consistency and minimize algorithm‐related discrepancies, both RadCalc and the TPS (RayStation) used the CCC algorithm for dose calculation. While known differences exist in the literature between CCC and Monte Carlo (MC) algorithms, especially in build‐up and surface regions, MC calculations were not used in this study.

## 3 RESULTS

### 3.1 Beam modeling for RadCalc software

**FIGURE 5:**
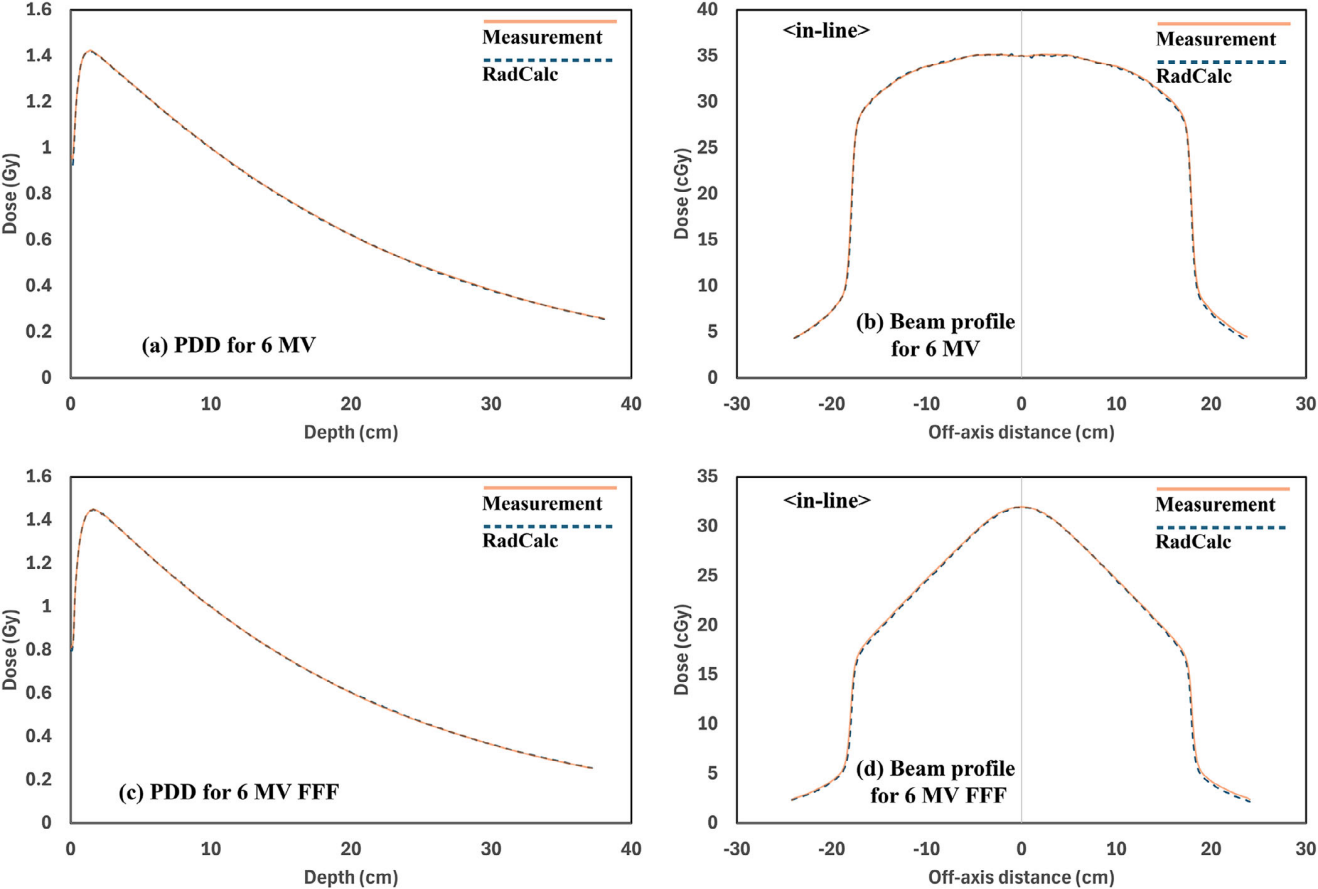
Beam modeling results comparing the measurement with RadCalc: (a) percentage depth dose (PDD) for 6‐MV, (b) beam profile (30 × 30 cm^2^, 10 cm depth) for 6‐MV, (c) PDD for 6‐MV flattening filter–free (FFF), and (d) beam profile (30 × 30 cm^2^, 10 cm depth) for 6‐MV FFF.

In this study, beam modeling in RadCalc was successfully conducted using measurement data already incorporated into our TPS. Our research team utilized SAD‐based measurement data embedded in the TPS. As the necessary field size data for the software had already been measured for TPS beam modeling, no additional measurements were required for RadCalc beam modeling. Figure 5 shows beam modeling results comparing the measurement data with RadCalc of PDD and beam profile (in‐line) data for 6‐MV and 6‐MV FFF photon beams used in RadCalc for beam modeling at a 30 × 30 cm^2^ field.

### 3.2 EPID modeling for RadCalc software

**FIGURE 6:**
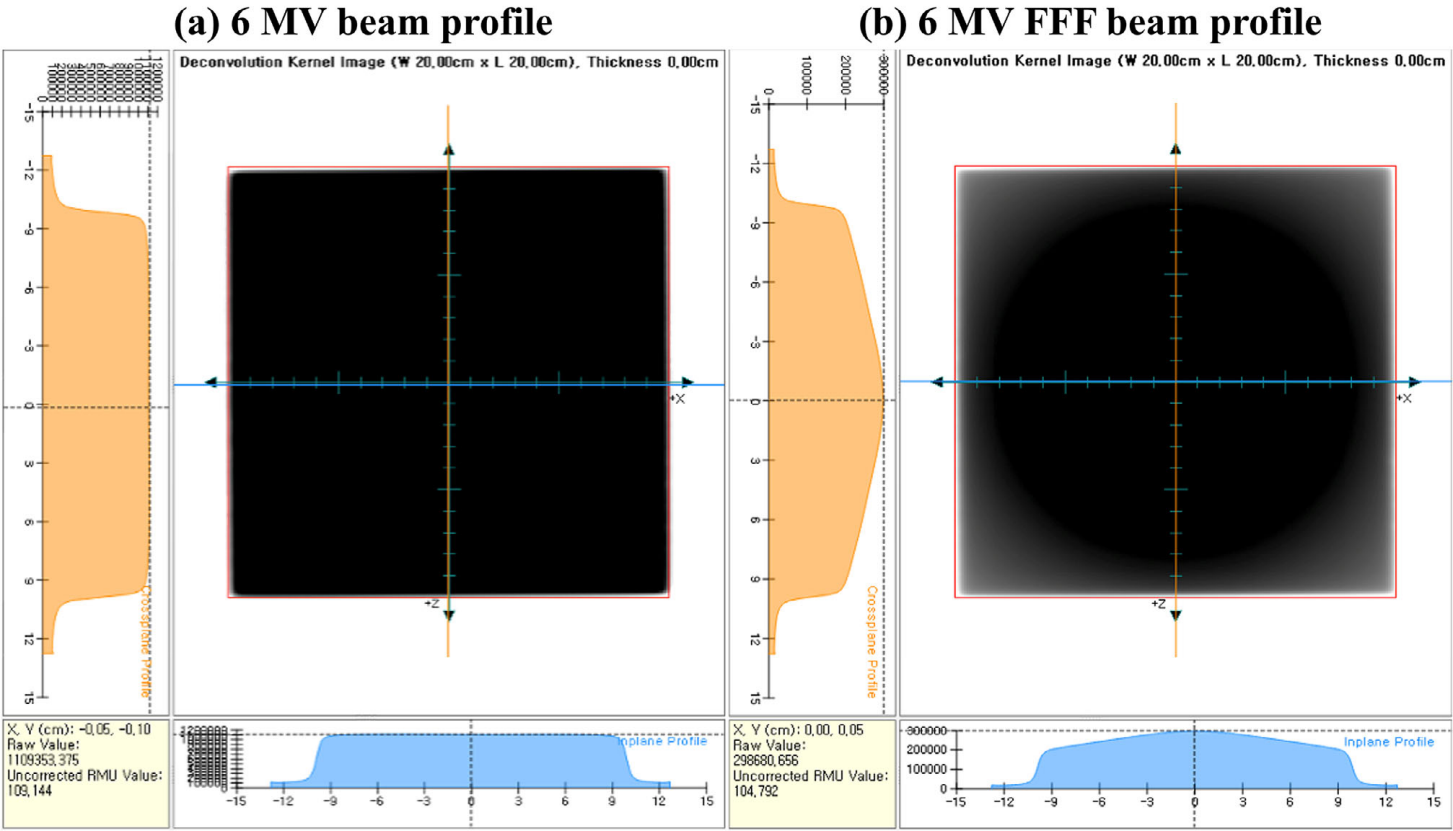
Beam profiles of the (a) 6‐MV and (b) 6‐MV flattening filter–free photon beams modeled using RadCalc software after the EPID modeling.

**FIGURE 7:**
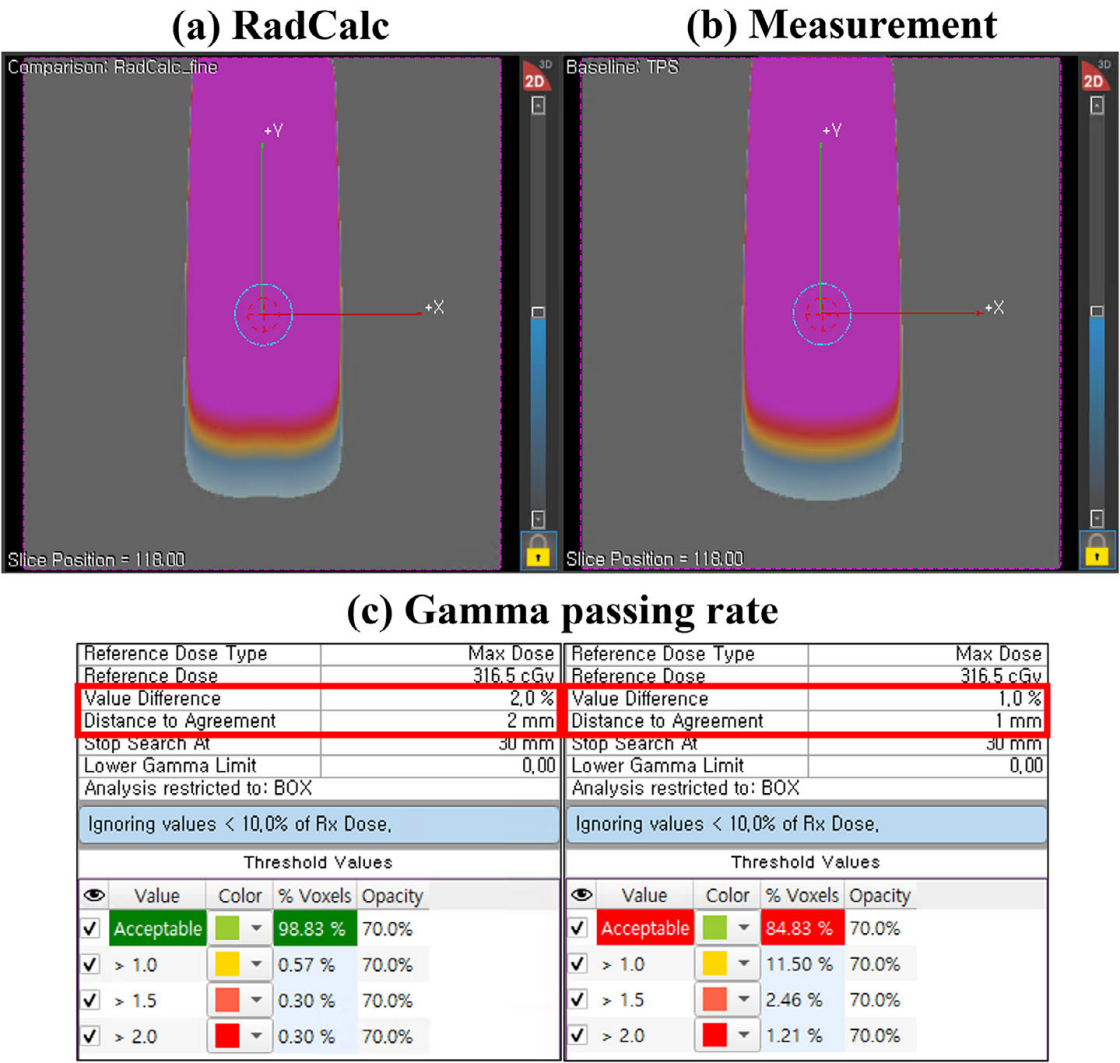
Dose comparison between (a) RadCalc and (b) measurements for a 20 × 20 cm^2^ field size of the 6‐MV photon beam. (c) Gamma passing rates at the 2%/2 mm and 1%/1 mm criteria.

Figure 6 shows EPID image data collected in a pretreatment setup for 6‐MV and 6‐MV FFF photon beams with a 20 × 20 cm^2^ field size. Figure 7 presents a dose distribution comparison between RadCalc (Figure 7(a)) and the measurement data (Figure 7(b)) for a 20 × 20 cm^2^ field size of the 6‐MV photon beam. Our evaluation, based on gamma analysis, revealed passing rates of 98.83% and 84.83% for the two distributions at 2%/2 mm and 1%/1 mm criteria, respectively (Figure 7(c)).

### 3.3 Validation of IDC

Table 1 shows the results of the RadCalc software calculation algorithm for 6‐MV and 6‐MV FFF photon beams. For 6‐MV plans, gamma passing rates ranged from 88.37% to 99.71% for the 3%/3 mm criterion and from 75.83% to 96.10% for the stricter 2%/2 mm criterion. Among the treatment sites analyzed, lung plans had the highest passing rates, with values reaching 99.71% (case 16) and 99.34% (case 17) under the 3%/3 mm evaluation standard. Conversely, breast plans exhibited comparatively lower passing rates, particularly under the 2%/2 mm criterion, with the lowest value recorded at 75.83% (case 4). For 6‐MV FFF plans, the gamma passing rates remained high, ranging from 95.32% to 99.59% under the 3%/3 mm criteria and from 85.73% to 99.13% under the 2%/2 mm criteria. The abdominal plan achieved the highest passing rate, reaching 99.59% under the 3%/3 mm and 97.26% under the 2%/2 mm criteria (case 28). Bone plans also showed high gamma passing rates, with all cases exceeding 95% for all cases under the 3%/3 mm criterion. However, case 22 had the lowest value of 85.73% under the 2%/2 mm criterion. Overall, under the 3%/3 mm criteria, gamma passing rates exceeded 90% in all cases for 6‐MV FFF, whereas for 6‐MV plans, only three cases failed to achieved this threshold. Under the stricter 2%/2 mm criterion, only six cases for 6‐MV plans exceeded a 90% passing rate, whereas for 6‐MV FFF, all but one case met this threshold. Notably, lung and liver plans consistently showed higher gamma passing rates between TPS and RadCalc, whereas breast plans had more significant variability, particularly under the 2%/2 mm gamma criterion.

**TABLE 1:** Results of the electronic portal imaging device (EPID) algorithm validations for 6‐MV and 6‐MV FFF photon beams comparing the gamma rates of the TPS and RadCalc under the 2%/2 mm and 3%/3 mm criteria for each treatment plan.

		Gamma passing rate (TPS vs. RadCalc, >90%)			
		6‐MV		6‐MV FFF	
No.	Site	3%/3 mm	2%/2 mm	3%/3 mm	2%/2 mm
1	Breast	93.05%	85.51%	–	–
2		92.49%	81.86%	–	–
3		89.96%	78.85%	–	–
4		88.37%	75.83%	–	–
5		89.30%	78.04%	–	–
6		95.52%	87.74%	–	–
7	Bone	95.44%	89.37%	–	–
8		96.90%	92.05%	–	–
9		95.58%	89.26%	–	–
10		96.47%	88.84%	–	–
11		98.51%	92.37%	–	–
12	Brain	95.88%	85.79%	–	–
13		97.62%	86.90%	–	–
14	Lymphatic node	96.60%	89.20%	–	–
15		93.77%	86.18%	–	–
16	Lung	99.71%	94.50%	–	–
17		99.34%	93.79%	–	–
18	Spleen	98.20%	96.10%	–	–
19	Liver	99.00%	96.04%	–	–
20	Bone	–	–	98.57%	93.62%
21		–	–	98.84%	96.32%
22		–	–	95.32%	85.73%
23		–	–	99.08%	95.21%
24	Lung	–	–	99.39%	96.17%
25		–	–	98.99%	94.81%
26	Prostate	–	–	99.14%	96.62%
27	Liver	–	–	99.52%	99.13%
28	Abdominal wall	–	–	99.59%	97.26%
29	Kidney	–	–	99.48%	97.19%

### 3.4 EPID‐based PSQA results

Tables 2 and 3 show the results of the PSQA for VMAT plans using the RadCalc software for 6‐MV and 6‐MV FFF photon beams. For the 6‐MV plans, a total of six cases failed to meet at least one of the criteria for either gamma passing rate or point‐dose evaluation (cases 1, 2, 3, 14, 16, and 17). For the 3%/3 mm criterion, the gamma passing rates between the TPS and EPID exceeded 90% for all plans except for cases 2 and 3 (breast case), with 86.78% and 89.52 %), case 14 (lymphatic node case), with 82.33%, and case 16 (lung case), with 88.97%. In the point‐dose measurement, the differences between the measured values and the TPS were within 5% for all plans except case 14, which had a difference of 5.71%, slightly exceeding the 5% criterion. This case involved a lymphatic node plan with complex MLC modulation and off‐axis target location, likely contributing to the discrepancy. Case 15, also a lymphatic node case, demonstrated acceptable gamma results but slightly elevated point dose differences. In case 11 (bone site), the measured dose was 3.44% higher than the TPS‐calculated dose, likely due to localized dose gradient effects near the chamber position. However, the RadCalc result demonstrated only a 0.30% difference from the measurement, indicating reliable performance of the EPID‐based calculation in this case. For the comparison between the measured values and the RadCalc software, the percentage difference was within 5% for all plans except for case 1 (5.10%) and case 17 (−5.13%).

**TABLE 2:** PSQA results for VMAT plans using RadCalc software for 6‐MV photons, including gamma passing rates for the 3%/3 mm and 2%/2 mm criteria, as well as point dose percentage differences between measurements, TPS, and RadCalc.

		Gamma passing rate (TPS vs. EPID, >90%)		Point dose % difference (<5%)	
No.	Site	3%/3 mm	2%/2 mm	Ion chamber vs. TPS	Ion chamber vs. RadCalc
1	Breast	99.88%	98.67%	3.43%	5.10%
2		86.78%	61.66%	3.98%	4.10%
3		89.52%	61.02%	4.46%	4.01%
4		93.85%	76.49%	2.49%	0.73%
5		95.39%	79.68%	1.56%	0.52%
6		97.33%	78.61%	2.15%	4.02%
7	Bone	99.05%	85.91%	1.77%	1.70%
8		99.60%	95.65%	1.92%	1.59%
9		98.34%	89.94%	2.97%	−0.34%
10		99.83%	94.59%	2.18%	0.36%
11		95.44%	88.59%	3.44%	0.30%
12	Brain	92.46%	61.68%	4.23%	3.80%
13		99.61%	98.59%	3.87%	3.64%
14	Lymphatic node	82.33%	67.21%	5.71%	1.85%
15		95.94%	81.47%	3.98%	2.58%
16	Lung	88.97%	53.61%	2.38%	−0.55%
17		99.16%	93.89%	2.80%	−5.13%
18	Spleen	99.39%	90.86%	1.36%	0.15%
19	Liver	96.70%	84.48%	0.25%	1.01%

**TABLE 3:** PSQA results for VMAT plans using RadCalc software for 6‐MV FFF photons, including gamma passing rates for the 3%/3 mm and 2%/2 mm criteria, as well as point dose percentage differences between measurements, TPS, and RadCalc.

		Gamma passing rate (TPS vs. EPID, >90%)		Point dose % difference (<5%)	
No.	Site	3%/3 mm	2%/2 mm	Ion chamber vs. TPS	Ion chamber vs. RadCalc
20	Bone	98.10%	90.85%	2.86%	−3.25%
21		98.99%	93.28%	1.89%	−2.19%
22		94.07%	83.24%	1.58%	−2.48%
23		99.56%	95.70%	3.14%	−2.27%
24	Lung	98.70%	94.39%	1.67%	−1.80%
25		95.07%	75.06%	1.50%	−3.25%
26	Prostate	98.61%	93.83%	1.42%	1.08%
27	Liver	98.76%	92.84%	0.26%	−0.06%
28	Abdominal wall	99.87%	95.66%	2.43%	−2.13%
29	Kidney	99.08%	86.00%	0.89%	−0.58%

In the case of the 6‐MV FFF, all cases satisfied the 3%/3 mm with gamma criterion, unlike the 6‐MV results. The 2%/2 mm criterion was also met for all but three cases (cases 22, 25, and 29). Specifically, for the 3%/3 mm criterion, case 22 had the lowest passing rate at 94.07%, whereas the other cases had high passing rates of approximately 99%. Regarding the point‐dose comparison, the differences between the measured values and TPS were primarily within 3%, indicating good agreement. Similarly, the comparison between the measured values and the RadCalc software showed differences within 3% for most cases, confirming consistent and reliable results.

## 4 DISCUSSION

In IDC, excluding EPID, beam modeling and machine parameters are critical determinants of accuracy in calculation‐based PSQA systems. Gamma analysis of beam modeling revealed an excellent passing rate of approximately 98% under the 2%/2 mm criterion, indicating minimal beam modeling errors in RadCalc. Given that our institution uses a standard criterion of at least 3%/3 mm, analysis results under this threshold can be considered reasonably reliable. All but three breast cases achieved high passing rates under the 3%/3 mm criterion, further supporting the superior calculation capability of the IDC system (Table 1). The three cases with gamma passing rates of approximately 90% were identified as breast plans in which the maximum dose was located off‐axis rather than at the isocenter. To address this, we tested modifications to adjustable MLC parameters (e.g., radiation light field offset) within the RadCalc software. However, these adjustments did not significantly improve the gamma passing rates in these cases.

When EPID data were incorporated into the analysis, four cases—two breast cases, one lung case, and one lymphatic node case—failed to achieve a gamma passing rate of 90% under the 3%/3 mm criterion. Among these, breast cases were expected to yield lower results due to inherent IDC limitations. Conversely, lung cases achieved passing rates of approximately 90%, indicating that the discrepancies were primarily related to calculation accuracy. Notably, case no. 14 (lymphatic node) showed a significantly low gamma passing rate (<90%) in RadCalc, confirming its failure. This finding aligned with the measurement‐based PSQA, where the point‐dose differences exceeded 5%, indicating poor plan deliverability.

For point‐dose comparison, breast cases exhibited higher discrepancies of approximately 4%–5% relative to measurement data, whereas lung case showed deviations exceeding −5%. The higher errors observed in breast cases are likely due to off‐axis planning, where the isocenter does not coincide with the maximum dose location. In such cases, ArcCHECK measurements are typically conducted in regions with steep dose gradients, potentially positioning the A1SL ion chamber in areas of high‐dose variation. In measurement‐based PSQA, recalibration and additional measurements may sometimes be performed at the isocenter to enhance accuracy, based on the clinical setup or dose gradient conditions. However, calculation‐based PSQA does not require such adjustments, as it assesses the entire 3D dose distribution. This approach facilitates a more direct comparison with the planned maximum dose location, simplifying the process relative to measurement‐based PSQA. Nevertheless, it is essential to recognize the limitations of IDC methods for off‐axis plans and to account for equipment specifications when interpreting PSQA results. The errors observed in lung cases are likely attributable to the influence of small fields, for which output calibration in EPID remains a persistent challenge; these limitations are not specific to RadCalc but are commonly encountered across other calculation‐based PSQA systems, necessitating user awareness and caution.^39^, ^40^, ^41^, ^42^

To address this, our institution routinely performs measurement‐based dose evaluation for cases involving small target sizes using tools such as the PinPoint 3D chamber (PTW, Germany). Additionally, the output stability of the EPID system integrated with the Harmony Pro LINAC was assessed. Over the 6‐month study period, daily noon‐time output measurements showed fluctuations within ±1%. This consistency confirms the reliability of the EPID‐based software for dose evaluation and calibration, ensuring accurate PSQA processes.

A key limitation of this study is that dose calculations were performed by mapping treatment plans onto the ArcCHECK phantom and comparing the calculated doses with measured values. However, directly comparing gamma analysis results is challenging because ArcCHECK primarily assesses fluence based on its rounded detector array, whereas this study utilized a comprehensive 3D dose gamma analysis. This fundamental difference may introduce discrepancies between the two evaluation methods. Moreover, inhomogeneity effects were not explicitly assessed, as the ArcCHECK phantom did not fully replicate the heterogeneous conditions of human tissues. Enhancing the precision of inhomogeneity evaluations would likely require improvements in dose calculation algorithms, such as MC‐based calculations. Although the CCC algorithm in RadCalc supports accurate dose calculations, its performance was not specifically evaluated in this study, nor were the MC algorithm capabilities within RadCalc tested. Additionally, beam modeling in this study was not rigorously constrained, aligning with the 1%/1 mm criterion, which resulted in an accuracy of approximately 85%. This limitation may have affected the evaluation of a few cases against the 2%/2 mm criterion gamma passing rate. Future studies should be conducted to refine beam modeling to improve accuracy and enhance the overall performance of RadCalc in VMAT plan evaluations. Nonetheless, the findings suggest that RadCalc remains clinically applicable under the conditions of our institution.

## 5 CONCLUSIONS

In this study, we aimed to share our experiences regarding the clinical setup, implementation process, and key considerations for integrating the RadCalc software with the Elekta Harmony Pro LINAC system into clinical practice. For the 6‐MV photon beam, most cases met the 3%/3 mm gamma criterion, confirming clinical applicability. In the case of 6‐MV FFF photon beams, all cases passed the 3%/3 mm criterion. Depending on beam modeling accuracy, a stricter 2%/2 mm criterion may also be achievable.

Despite these findings, the RadCalc software is deemed clinically sufficient due to its ability to perform 3D dose reconstruction using EPID data, enabling intuitive and direct treatment plan verification using the Harmony Pro system. Although this study used the ArcCHECK phantom to eliminate patient‐specific information, we anticipate that using RadCalc with actual patient CT images can facilitate dose calculations tailored to individual patient anatomy.

## AUTHOR CONTRIBUTIONS

D. H. Yoo, H. G. Lee, S. M. Lee, S. R. Han, T. H. Kim, C. H. Kim, M. C. Han, and J. S. Kim contributed to the study conception and design. D. H. Yoo and H. G. Lee conducted data collection. D. H. Yoo conducted the statistical analyses. D. H. Yoo and M. C. Han drafted and revised the manuscript. M. C. Han verified the authenticity of all raw data. All authors revised and approved the final manuscript.

## CONFLICT OF INTEREST STATEMENT

Our institution is conducting joint research with LAP.

## Data Availability

The datasets generated during the current study will be available from corresponding author on reasonable request.
